# Postbiotic pA1c^®^HI for Preventing Insulin Resistance and Obesity in a *Caenorhabditis elegans* Model of Prediabetes

**DOI:** 10.3390/ijms26168094

**Published:** 2025-08-21

**Authors:** Deyan Yavorov-Dayliev, Iñaki Iturria, Leyre Iriarte, Miriam Araña, Miguel Barajas, Josune Ayo

**Affiliations:** 1 Genbioma Aplicaciones S.L., Cein Centro Europeo, Office D3, Pl. Cein, 1-5, Pol. Ind. Mocholí, 31110 Noáin, Navarra, Spain; iiturria@genbioma.com (I.I.); liriarte@genbioma.com (L.I.); josune@genbioma.com (J.A.); 2 Biochemistry Area, Health Science Department, Faculty of Health Sciences, Public University of Navarra, 31008 Pamplona, Navarra, Spain; miriam.arana@unavarra.es (M.A.); miguel.barajas@unavarra.es (M.B.)

**Keywords:** postbiotics, probiotics, obesity, diabetes, metabolic syndrome, *C. elegans*

## Abstract

Cardiometabolic diseases such as obesity, prediabetes (PreD), and type 2 diabetes (T2D) are global health challenges linked to metabolic dysfunction. While probiotics show promise, postbiotics offer advantages in stability, safety, and food incorporation. This study evaluates the postbiotic pA1c^®^HI, a heat-inactivated form of the probiotic pA1c^®^, for its potential in modulating glucose and lipid metabolism in *Caenorhabditis elegans*, compared to its live form. Worms were supplemented with pA1c^®^HI and live pA1c^®^ in glucose-enriched media. Fat accumulation, gene expression, oxidative stress, and lifespan were measured using Nile Red and DHE staining, qPCR, and longevity assays. pA1c^®^HI significantly reduced glucose-induced fat accumulation, achieving fat reduction comparable to the anti-obesity drug orlistat and showing superior efficacy compared to the live probiotic form. It modulated the expression of genes associated with lipid oxidation (*acox-1*, *cpt-2*), fatty acid synthesis (*fat-5*), insulin signaling (*daf-2*, *daf-16*), and oxidative stress response (*skn-1*). Synergistic combinations with chromium picolinate (PC) and zinc (Zn) further enhanced metabolic outcomes. Importantly, pA1c^®^HI retained efficacy after thermal treatment (121–135 °C), supporting its potential for use in processed foods. pA1c^®^HI is a stable, effective postbiotic that modulates key pathways associated with obesity, PreD, and T2D in *C. elegans*, with superior performance to the live probiotic and added benefits when combined with PC and Zn.

## 1. Introduction

Obesity, prediabetes (PreD) and type 2 diabetes (T2D) are major global health concerns [1], with rising incidence rates contributing to an increase in chronic diseases such as cardiovascular disorders [2], hypertension [3], and dyslipidemia [4]. The three conditions are primarily driven by disruptions in glucose and lipid metabolism. While probiotic interventions have shown promise in addressing metabolic imbalances [5], growing scientific interest is now focused on postbiotics, non-viable microbial cells, or their components [6], which may offer equal or superior benefits with improved safety, versatility, and ease of incorporation into food products [7].

Postbiotics retain bioactive microbial compounds such as short-chain fatty acids (SCFAs), enzymes, and peptides without requiring viability to exert functional effects [8,9]. This inactivation process enhances thermal and shelf stability, allowing for integration into food products subjected to industrial heat treatments such as pasteurization or Ultra-High-Temperature (UHT) processes [10,11]. Additionally, unlike live probiotics, postbiotics eliminate concerns related to bacterial viability, overgrowth, or potential side effects in immunocompromised individuals [12]. These properties position postbiotics as safe, stable, and scalable candidates for long-term therapeutic interventions.

In our study, we investigated the potential of the postbiotic pA1c^®^HI to modulate obesity, PreD, and T2D using the model organism *Caenorhabditis elegans*. This nematode offers a valuable in vivo system for metabolic studies, as it shares conserved metabolic pathways with humans, including insulin and lipid metabolism regulation [13]. The primary goal of our research was to evaluate whether pA1c^®^HI, a heat-killed derivative of the probiotic pA1c^®^, could exert superior metabolic benefits compared to its live probiotic form, particularly in the context of obesity and T2D.

Previous studies of our research group [14,15,16,17,18,19,20] have demonstrated that the live probiotic pA1c^®^ improves key metabolic parameters, such as reducing fat accumulation and improving insulin sensitivity and glucose regulation in both *C. elegans* and mammalian models. However, emerging data, including our own, suggest that the heat-inactivated form may preserve or concentrate bioactive compounds, potentially enhancing efficacy on glucose and lipid metabolism via direct host signaling modulation. Thus, this study aimed to provide new insights into the therapeutic potential of postbiotics, specifically pA1c^®^HI, in managing obesity and T2D. By comparing its effects to the live probiotic pA1c^®^, we seek to establish a more effective, stable, and scalable strategy for addressing the growing burden of metabolic disease.

## 2. Results

### 2.1. pA1c^®^HI Postbiotic Counteracts Glucose-Induced Fat Accumulation with Effectiveness Comparable to the Anti-Obesity Drug Orlistat

**Figure 1:**
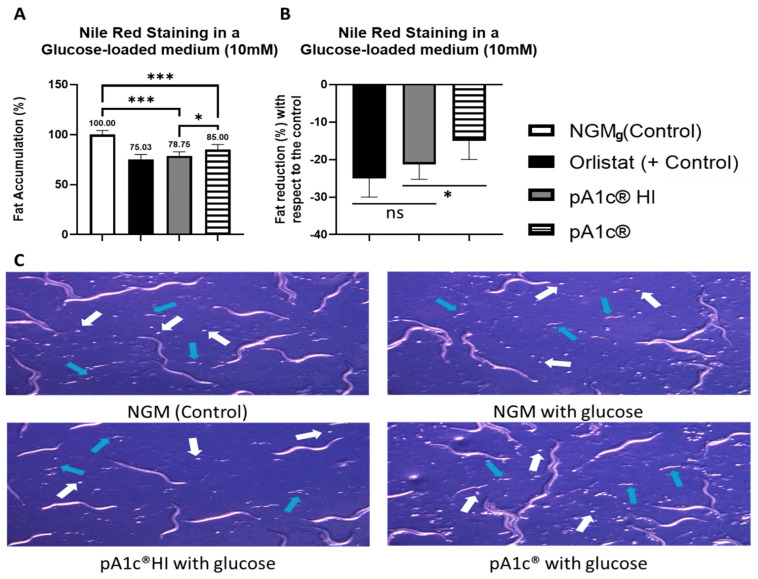
Nile Red staining of pA1c^®^HI- and pA1c^®^-supplemented worms in a glucose-loaded (10 mM) medium. (**A**) Percentage of fat accumulation with respect to the control. (**B**) Percentage of fat reduction with respect to the control. (**C**) Microscope observation of the presence of eggs (white arrows) and L1 larvae (blue arrows). * *p* < 0.05; *** *p* < 0.001, ^ns^ *p* > 0.05.

The measurement of fat storage and lipid droplets in *C. elegans* is possible by the visualization under microscopy of fat-soluble dyes such as Nile Red [21]. The following results were obtained in an experiment with a sample size of 80 worms per group, ensuring robust and reliable data. At a dose of 5 × 10^6^ cells/mL, pA1c^®^HI significantly counteracted the effect of glucose and reduced fat accumulation in N2 wild-type *C. elegans* by 21.2 ± 5.0% compared to control worms (*p* < 0.001) (Figure 1A,B). Remarkably, the fat-reducing efficacy of pA1c^®^HI at 5 × 10^6^ cells/mL was comparable to that of the anti-obesity drug orlistat (25.0 ± 7.0% fat reduction), as no significant differences were observed between them (*p* > 0.05) in comparison with control worms (Figure 1A,B).

Additionally, pA1c^®^HI demonstrated greater efficacy in counteracting the effect of glucose and reducing fat accumulation compared to the probiotic pA1c^®^. At the same dose of 5 × 10^6^ cells/mL, pA1c^®^HI reduced fat storage by 21.2 ± 5.0%, whereas worms supplemented with 5 × 10^6^ CFU/mL of pA1c^®^ showed only a 15.0 ± 8.0% reduction in fat accumulation (*p* < 0.05) (Figure 1A,B).

Moreover, the glucose-counteracting activity of pA1c^®^HI did not present any detrimental effect on the organisms’ growth and correct development. Comparing pA1c^®^HI-supplemented worms with control and pA1c^®^-supplemented worms at 72 h, it was observed that all the groups exhibited the presence of eggs and L1 larvae as expected, with no differences in their time of appearance (Figure 1C).

### 2.2. pA1c^®^HI Regulates the Insulin Signaling Pathway, Inhibits Fatty Acid Biosynthesis, and Activates the Fatty Acid β-Oxidation Process

**Figure 2:**
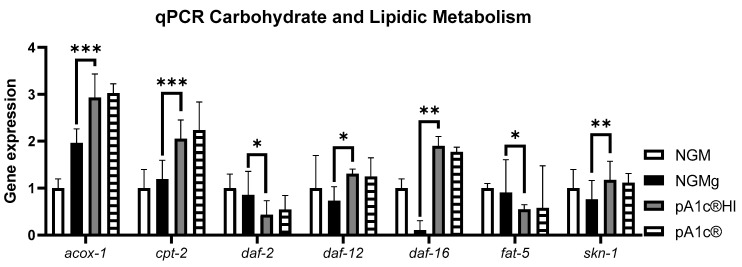
Gene expression analysis quantified by real-time PCR (qPCR) in *C. elegans*. Gene expression levels were normalized to the housekeeping gene (*pmp-3*). Data are expressed using the 2^−∆∆Ct^ method: * *p* < 0.05; ** *p* < 0.01; *** *p* < 0.001.

The qPCR analysis of glucidic and lipidic metabolism genes in *C. elegans* revealed that the postbiotic pA1c^®^HI was significantly more effective than the control group in nematode growth medium (NGM) in modulating key metabolic pathways (*p* < 0.001). Regarding lipid metabolism, the postbiotic significantly increased the peroxisomal (*acox-1*) and mitochondrial (*cpt-2*) beta-oxidation process (*p* < 0.001) by 50%, in addition to inhibiting (>50%) fatty acid biosynthesis (*fat-5*) (*p* < 0.05), in comparison with the NGMg group. Concerning glucidic metabolism, pA1c^®^HI significantly decreased the expression of homolog insulin receptor in *C. elegans daf-2* (*p* < 0.05) and significantly increased the expression of *daf-16* (the key gene of the insulin signaling pathway in *C. elegans*) by 20 times compared to NGMg (*p* < 0.01). In addition, in reference to other metabolic pathways, pA1c^®^HI significantly increased the expression of *daf-12* (longevity and cholesterol regulation) and *skn-1* (lifespan and oxidative stress) compared to NGMg (*p* < 0.05 for *daf-12* and *p* < 0.01 for *skn-1*). While the probiotic pA1c^®^ also showed some effects, the trends indicate higher efficacy for pA1c^®^HI across all analyzed genes (Figure 2).

**Figure 3:**
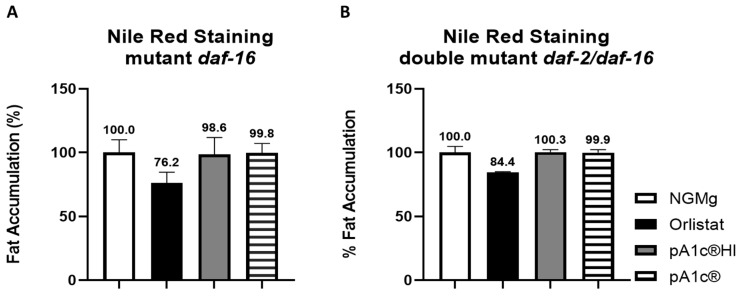
Nile Red staining of pA1c^®^HI- and pA1c^®^-supplemented worms in a glucose-loaded (10 mM) medium. (**A**) *daf-16* mutant strain. (**B**) *daf-2/daf-16* double mutant strain.

To investigate the role of the insulin signaling pathway in pA1c^®^HI glucose-counteracting and fat-reducing effects, mutants of *daf-16* and *daf-2*/*daf-16* strains were examined after being exposed to the postbiotic. Nile Red staining revealed that the *daf-16* mutation did not reverse the glucose-counteracting and body-fat-reducing effect of pA1c^®^HI (Figure 3A). Additionally, it was found that supplementation with pA1c^®^HI did not reduce fat accumulation in the *daf-2/daf-16* double mutant (Figure 3B) nor in *C. elegans*. The same results were observed with the probiotic intervention.

### 2.3. pA1c^®^HI Counteracts the High-Glucose-Induced Nuclear Localization of daf-16 More Effectively than the Alive pA1c^®^

**Figure 4:**
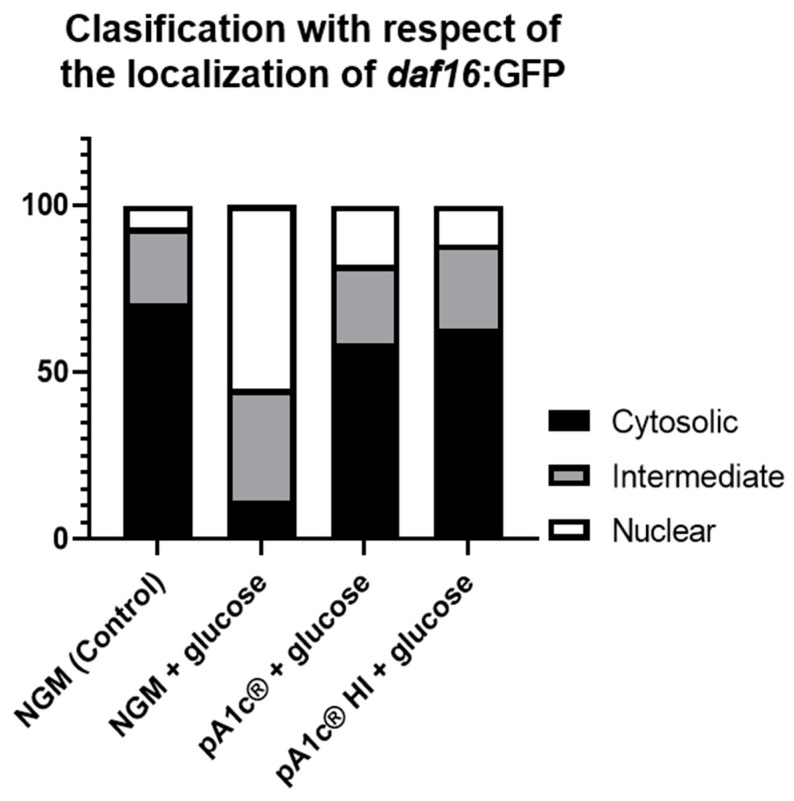
Classification of worms with respect to *daf-16*: GFP localization.

In control conditions, a higher cytosolic expression of the gene *daf-16* was observed in *C. elegans* grown in NGM plates. Upon the addition of glucose (10 mM) to the medium, a nuclear translocation of *daf-16* was observed. Supplementation with pA1c^®^HI or pA1c^®^ reversed the glucose-induced nuclear translocation of *daf-16*, reaching control values. Remarkably, this effect was more effective (>6%, *p* < 0.05) in the pA1c^®^HI group than in the pA1c^®^ group (Figure 4).

### 2.4. The Postbiotic pA1c^®^HI Enhances Oxidative Stress Response and Lifespan

**Figure 5:**
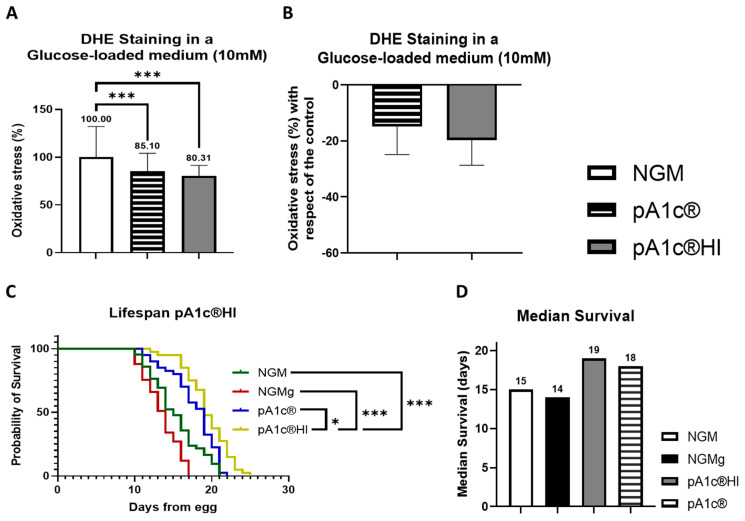
ROS production quantification (measured by DHE) and lifespan assay of pA1c^®^HI- and pA1c^®^-supplemented worms in a glucose-loaded (10 mM) medium. (**A**) Percentage of ROS with respect to the control. (**B**) Percentage of ROS reduction with respect to the control. (**C**) Lifespan analyses of control, postbiotic, and probiotic worms. (**D**) Median survival (days). * *p* < 0.05, *** *p* < 0.001.

Concerning oxidative stress of the worms, the postbiotic significantly reduced ROS accumulation in *C. elegans* by 19.7 ± 9.8% compared to the control group (*p* < 0.001) in NGMg media. Although the probiotic pA1c^®^ also reduced ROS levels (−14.9 ± 12.1%), the high variability in this response limits the reliability and consistency of the effect, suggesting that the postbiotic form offers a more robust and reproducible alternative (Figure 5A,B).

Moreover, at a dose of 5 × 10^6^ cells/mL, the postbiotic significantly increased the longevity and life expectancy of *C. elegans* by 26.5% (4 days of median survival) compared to the control group (*p* < 0.001), 36.0% (5 days of median survival) compared to the high-glucose group (*p* < 0.001), and 6.0% (1 day of median survival) compared to the probiotic group (*p* < 0.05) (Figure 5C,D).

### 2.5. Heat Stability of pA1c^®^HI

**Figure 6:**
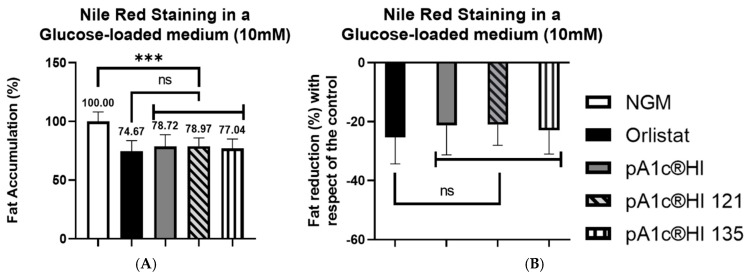
Nile Red staining of pA1c^®^HI-supplemented worms at different temperatures (121–135 °C). (**A**) Percentage of fat accumulation compared to the control. (**B**) Percentage of fat reduction compared to the control. *** *p* < 0.001, ^ns^ *p* > 0.05.

The postbiotic pA1c^®^HI subjected to 121 °C and 135 °C for 30 min significantly reduced fat accumulation in *C. elegans* by 21–23% compared to the control group (*p* < 0.001) in a glucose-loaded (10 mM) medium (Figure 6). Notably, no statistically significant differences were observed between the temperature-treated pA1c^®^HI and the positive control, orlistat. Furthermore, the trial demonstrated that the postbiotic exposed to these high temperatures retained the same efficacy in glucose and lipid metabolisms as the untreated pA1c^®^HI.

### 2.6. Synergistic Effects of pA1c^®^HI Combined with Chromium Picolinate and Zinc on Glucose Counteraction and Fat Reduction

**Figure 7:**
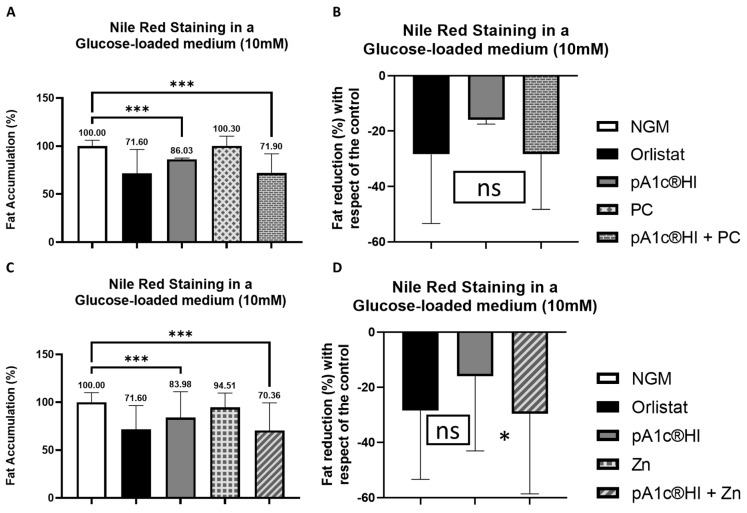
Nile Red staining of the combination pA1c^®^HI + chromium-supplemented worms. (**A**) Percentage of fat accumulation compared to the control. (**B**) Percentage of fat reduction compared to the control. Nile Red staining of the combination pA1c^®^HI + zinc-supplemented worms. (**C**) Percentage of fat accumulation compared to the control. (**D**) Percentage of fat reduction compared to the control. * *p* < 0.05; *** *p* < 0.001, ^ns^ *p* > 0.05.

Figure 7 showed that the postbiotic significantly counteracted glucose and reduced fat accumulation in *C. elegans* by over 14.0–16.0% compared to the control group (*p* < 0.001) with glucose in the media (Figure 7). Moreover, the combination of the postbiotic with chromium picolinate (PC) enhanced the glucose-counteracting and fat-reducing effects, achieving a 29.0% reduction in glucose-induced fat accumulation in comparison with control worms (Figure 7A,B). Notably, no statistically significant differences were observed between either the postbiotic alone or the postbiotic–PC combination and the fat-reducing drug orlistat. Additionally, the combination of the postbiotic with zinc (Zn) further improved the glucose-counteracting and the fat-reducing effects, achieving a 30.0% reduction in glucose-induced fat accumulation (Figure 7C,D) in comparison with control nematodes. As in the case of the combination with PC, no statistically significant differences were observed between either the postbiotic alone or the postbiotic–Zn combination and the positive control, orlistat. Nevertheless, a significant difference and, in turn a synergy were indeed observed between the combination containing pA1c^®^HI and zinc and the postbiotic alone (*p* < 0.05).

### 2.7. Synergistic Effects of pA1c^®^HI Combined with Chromium Picolinate and Zinc on Longevity and Lifespan

**Figure 8:**
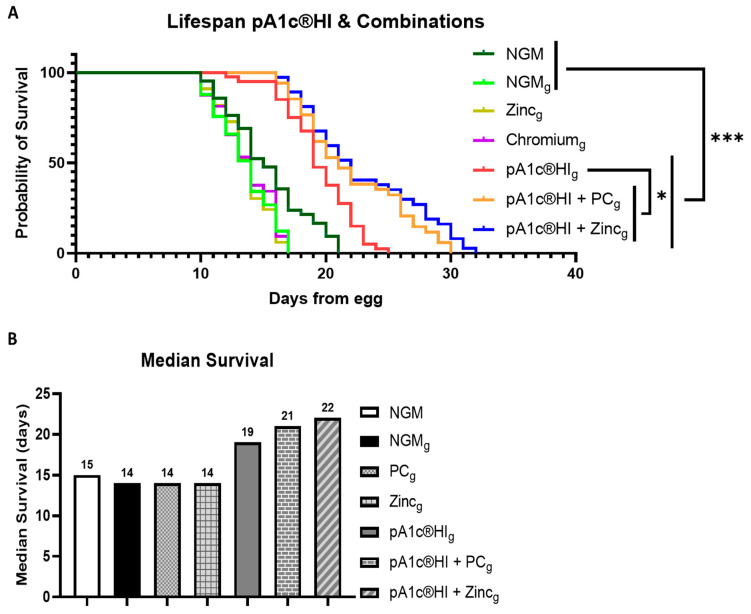
(**A**) Lifespan analyses. (**B**) Median survival (days). * *p* < 0.05, *** *p* < 0.001.

Lifespan analysis revealed that supplementation with pA1c^®^HI significantly increased by 36% (5 days) the longevity of *C. elegans* compared to the glucose control group NGMg (*p* < 0.001). Furthermore, the combination of pA1c^®^HI with zinc (pA1c^®^HI + zinc) and pA1c^®^HI with chromium picolinate (pA1c^®^HI + PC) extended the lifespan even further (57%, or 8 days, and 50%, or 7 days, respectively) in comparison with control glucose worms. In addition, both the combination of pA1c^®^HI + zinc and the combination of pA1c^®^HI + PC showed a statistically significant improvement compared to pA1c^®^HI alone (*p* < 0.05), indicating a synergistic effect between the components of the combinations (Figure 8A). In contrast, treatments with zinc or chromium alone did not significantly differ from the glucose control group. Figure 8B further confirms the observed synergy, as the median survival of *C. elegans* treated with pA1c^®^HI + zinc and pA1c^®^HI + PC was markedly higher compared to all other groups, including the control and individual treatments.

### 2.8. Combinations Containing pA1c^®^HI Modulated Glucose and Lipid Metabolism in a More Effective Way than pA1c^®^HI Alone

**Figure 9:**
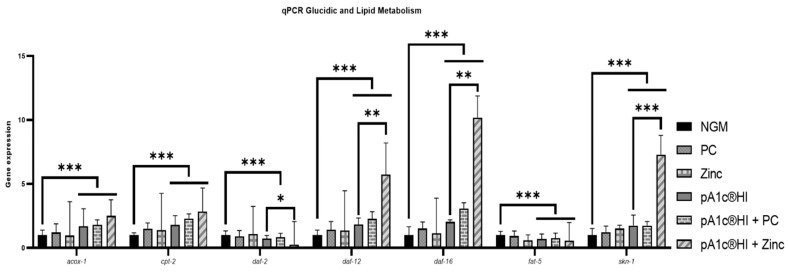
Gene expression analysis quantified by real-time PCR (qPCR) in *C. elegans*. Gene expression levels were normalized to the housekeeping gene (*pmp-3*). Data are expressed using the 2^−∆∆Ct^ method: * *p* < 0.05; ** *p* < 0.01; *** *p* < 0.001.

Gene expression analyses in a hyperglycemic media were performed (Figure 9). Regarding lipid metabolism, the postbiotic alone showed that compared with control worms, it increased the expression of *acox-1* (peroxisomal β-oxidation) and *cpt-2* (mitochondrial β-oxidation) by 70.0% and 81.0%, respectively (*p* < 0.001). The combination of the postbiotic with PC further enhanced these effects, significantly increasing both gene expressions by 79.0% and 129.0%, respectively (*p* < 0.001). Moreover, the postbiotic + Zn treatment had the most pronounced effect, significantly overexpressing *acox-1* by 152.0% and *cpt-2* by 184.0% (*p* < 0.001). Additionally, fatty acid biosynthesis (*fat-5*) was significantly inhibited across all postbiotic treatments, with the strongest suppression observed in the postbiotic + Zn group (−41.0% vs. NGM, *p* < 0.001).

Concerning carbohydrate metabolism, the postbiotic alone reduced *daf-2* expression by 27.0% (*p* < 0.001). The combination with PC also resulted in a downregulation of 16.0%, but the strongest inhibitory effect was observed in the postbiotic + Zn group, where *daf-2* expression was reduced by 73.0% compared to NGMg (*p* < 0.001) and significantly reduced by 50.0% compared with pA1c^®^HI alone (*p* < 0.05). Meanwhile, the postbiotic significantly increased *daf-16* expression by more than 100.0% (*p* < 0.001). This effect was further amplified by the postbiotic + PC treatment (+200.0%, *p* < 0.001) and dramatically enhanced in the postbiotic + Zn group, where *daf-16* expression significantly increased more than 10-fold compared with NGMg (*p* < 0.001) and more than 5-fold in comparison with the postbiotic alone (*p* < 0.01)

In addition, in relation to other metabolic and longevity-related pathways, the postbiotic significantly upregulated *daf-12* (+85.0%, *p* < 0.001) and *skn-1* (+75.0%, *p* < 0.001) compared to NGMg. The postbiotic + PC combination further increased *daf-12* expression (+127.0%, *p* < 0.001) and *skn-1* (+80.0%, *p* < 0.001), while the postbiotic + Zn supplementation resulted in a striking upregulation of both genes, with *daf-12* increasing by 6-fold and *skn-1* by 7-fold compared to NGM (*p* < 0.001 for both) and significantly increasing *daf-12* by 3-fold (*p* < 0.01) and *skn-1* by 4-fold (*p* < 0.001) compared with the postbiotic alone.

## 3. Discussion

*Caenorhabditis elegans*, a free-living nematode, is an experimental in vivo model that has been extensively employed in the study of obesity due to its favorable features such as its compact size, short life cycle, large brood size, easy handling, low cost, availability of complete genetic information, 65% conserved human diseases-associated genes, and relatively easy genetic manipulation [22]. The regulation of carbohydrate and lipid metabolism in *C. elegans* is closely interconnected, with both pathways influencing each other to maintain cellular energy balance [23]. Disruptions in glucose metabolism often impact lipid storage and vice versa, highlighting the conserved relationship between these metabolic processes. This interconnectedness reflects the shared regulatory mechanisms that govern energy homeostasis, demonstrating the importance of a coordinated metabolic response in *C. elegans*, which is also observed in higher organisms [24].

Our findings demonstrate that the postbiotic pA1c^®^HI has a notable potential to reduce glucose-induced fat accumulation in *C. elegans*, achieving efficacy comparable to that of the anti-obesity drug orlistat. This result is especially relevant in the context of our objective to evaluate the therapeutic potential of pA1c^®^HI in the management of obesity PreD and T2D, as it suggests that the metabolic benefits of this postbiotic may be in line with those of conventional drug treatments. Additionally, the higher efficacy of pA1c^®^HI in terms of its glucose-counteracting effect compared to its probiotic form, pA1c^®^, supports the hypothesis that heat inactivation enhances the biological activity of this compound, possibly through the concentration of bioactive metabolites responsible for metabolic modulation in the host. The lack of adverse effects on nematode growth and development further suggests that pA1c^®^HI may be a safe and effective metabolic intervention strategy.

In addition to metabolic modulation, growing scientific interest is now focused on the ability of postbiotics to exert immunomodulatory effects, improve gut barrier function, and modulate inflammation, which are all relevant in the context of obesity and T2D. This positions postbiotics like pA1c^®^HI as promising multifunctional ingredients.

Moreover, our results reveal that the metabolic benefits of pA1c^®^HI can be further enhanced when combined with specific bioactive compounds, such as PC and Zn. These combinations demonstrated a significant improvement in glucose-counteracting and fat-reducing effects. Notably, the pA1c^®^HI-Zn combination showed a significantly greater effect than pA1c^®^HI alone (*p* < 0.05), indicating a true synergistic interaction rather than a simple additive effect. This synergy suggests that the mechanisms of action of Zn complement those of pA1c^®^HI, potentially optimizing its impact on glucose and lipid metabolism.

Furthermore, the remarkable ability of the postbiotic to retain its glucose-counteracting and fat-reducing efficacy even after being subjected to pasteurization and UHT temperatures for 30 min underscores its potential as a robust and versatile tool in combating T2D and obesity. Unlike probiotics, which often require strict storage and handling conditions to maintain viability, pA1c^®^HI demonstrates exceptional stability, allowing it to be incorporated into a wide range of food matrices, including those subjected to high-temperature processes such as baking, cooking, or sterilization. This amazing resistance not only broadens the field of applications of pA1c^®^HI in functional foods and dietary interventions but also improves its shelf life and transportability. Its consistent efficacy in counteracting hyperglycemia and reducing fat accumulation, even after exposure to such high-temperature treatments, positions pA1c^®^HI as a superior alternative to probiotics, offering both practical advantages and potent bioactive benefits to promote health and address T2D- and obesity-related challenges.

In *C. elegans*, the insulin signaling pathway and other metabolic regulatory mechanisms (oxidative stress, lifespan, and cholesterol metabolism) are controlled by several key genes, including *daf-2*, *daf-16*, *daf-12*, and *skn-1*. The *daf-2* gene encodes the homolog of the human insulin/IGF-1 receptor [25] and plays a crucial role in modulating glucose and lipid metabolism [26]. Reduced *daf-2* expression leads to the activation of *daf-16*, a transcription factor that regulates genes involved in stress resistance, fat mobilization, and longevity [27]. Increased *daf-16* expression enhances energy utilization, promoting lipid oxidation and improving metabolic health [28]. Moreover, the *daf-12* gene encodes a nuclear hormone receptor that regulates developmental timing, longevity, and cholesterol metabolism [29]. Its activation is associated with improved lipid and cholesterol homeostasis and lifespan extension. On the other hand, *skn-1* is a transcription factor responsible for oxidative stress responses and lifespan regulation. It activates antioxidant defense mechanisms and promotes cellular stress resistance, contributing to overall metabolic health and longevity [30]. Regarding lipid metabolism and fat storage, *daf-16*, *acox-1*, *cpt-2*, and *fat-5* play central roles. The *daf-16* gene, a crucial transcription factor, is involved in controlling fat accumulation and mobilization. Activation of *daf-16* enhances fat utilization and reduces lipid storage, while its impaired function can lead to excessive fat accumulation, contributing to obesity [31]. Similarly, the *acox-1* gene encodes an enzyme involved in the initial steps of peroxisomal fatty acid oxidation, promoting the breakdown of fats for energy production [32]. *cpt-2* regulates the transport of fatty acids into mitochondria for oxidation, further supporting fat utilization [33]. The *fat-5* gene, which encodes a desaturase enzyme, influences the composition of fatty acids, affecting fat storage and membrane fluidity [34]. Together, these genes coordinate lipid metabolism, and their dysregulation can lead to an imbalance in fat storage, promoting obesity.

Our results indicate that pA1c^®^HI significantly modulated these key metabolic pathways, demonstrating a marked improvement in glucose and lipid homeostasis. The significant reduction in *daf-2* expression, accompanied by a 20-fold increase in *daf-16*, suggests that pA1c^®^HI enhances the regulation of insulin signaling, thereby potentially improving glucose utilization and energy homeostasis. Moreover, the synergy observed in the combination treatments with PC and Zn was also evident in the modulation of *daf-2* and *daf-16*, with the pA1c^®^HI + Zn combination showing a dramatic 50% reduction in *daf-2* expression and a more than 5-fold increase in *daf-16* expression compared with pA1c^®^HI alone. This shift in the *daf-2*/*daf-16* axis is a critical predictor of improved metabolic health, as *daf-16* activation is associated with increased lipid oxidation and reduced fat storage. Furthermore, increased expression of *daf-12* and *skn-1* suggests that pA1c^®^HI not only influences glucose and lipid metabolism but also promotes pathways related to cholesterol, longevity and resistance to oxidative stress. These findings are consistent with our initial hypothesis that postbiotics may exert wider metabolic benefits than fat reduction, potentially providing protection against age-related metabolic disorders. The fact that pA1c^®^HI showed higher efficacy than its probiotic homologue in modulating these genes lends even more support to the idea that the postbiotic form can provide a more stable and potent therapeutical alternative. In addition, the synergistic effects of pA1c^®^HI with PC and Zn led to a marked upregulation of *daf-12* and *skn-1*, particularly in the pA1c^®^HI + Zn combination, confirming our hypothesis. Finally, the combination of pA1c^®^HI with PC and Zn showed increased expressions of *acox-1* and *cpt-2*, both of which are essential for fatty acid oxidation. In addition, the remarkable inhibition of fatty acid biosynthesis (*fat-5*) in all postbiotic treatments, particularly in the pA1c^®^HI + Zn combination, further demonstrates the potent effect of pA1c^®^HI on lipid metabolism. The enhancement of the expression of these genes in the combined treatments indicates that the synergistic effect of these compounds significantly amplifies the lipid-lowering and glucose-counteracting benefits of pA1c^®^HI.

In addition to the observed effects on fat accumulation and glucose metabolism, the postbiotic pA1c^®^HI appears to modulate oxidative stress responses in *C. elegans*. Our results indicate that pA1c^®^HI reduces the accumulation of reactive oxygen species (ROS) and upregulates the expression of *skn-1*, a key regulator of antioxidant defenses, as well as *daf-12*, among other genes. Based on these findings, we hypothesize that supplementation with pA1c^®^HI may enhance the organism’s resilience to oxidative stress by reducing ROS levels through *skn-1*-mediated pathways, with possible involvement of *daf-16* as well. Furthermore, the increase in *daf-12* expression coupled with the significant reduction in fat accumulation suggests a potential improvement in cholesterol metabolism. Although cholesterol levels were not directly measured in this study, the close relationship between cholesterol and lipid metabolism in *C. elegans* makes this a plausible mechanism. This opens interesting avenues for future research and potential applications of pA1c^®^HI as a multifunctional postbiotic that not only targets metabolic dysfunction but also improves oxidative stress resilience and lipid homeostasis.

Regarding the insulin signaling null mutant assays, the fact that *daf-16* and *daf-2*/*daf-16* mutations completely abolished the glucose-counteracting and fat-reducing effects of pA1c^®^HI suggests that a functional insulin signaling pathway is essential for the mechanism of action of the postbiotic. Furthermore, the ability of pA1c^®^HI to counteract glucose-induced nuclear translocation of *daf-16* further supports its role in restoring metabolic balance. Under normal conditions, *daf-16* remains mostly cytosolic; however, upon exposure to glucose, its nuclear localization is triggered as part of a stress response. The reversal of this translocation by pA1c^®^HI suggests that the postbiotic mitigates the detrimental effects of hyperglycemia, helping to maintain insulin signaling and metabolic homeostasis. Notably, this effect was significantly more pronounced in worms supplemented with pA1c^®^HI than in those receiving the live probiotic, reinforcing the idea that the postbiotic form may exert a more potent regulatory effect. These results are in agreement with previous studies of our group highlighting *daf-16* as a key regulator of glucose and lipid metabolism in *C. elegans* [14,17].

Nevertheless, studies in other organisms have shown that the microbiota context, which is absent in *C. elegans*, can significantly influence the efficacy of microbial interventions. Thus, it remains important to assess postbiotic effects in systems where host–microbiota interactions are fully represented.

In *C. elegans*, the regulation of lipid metabolism is closely linked to both lifespan and aging [35], with obesity playing a significant role in these processes. Increased fat accumulation, often associated with impaired lipid metabolism, has been shown to shorten the lifespan of *C. elegans* [36]. This is because excessive lipid storage can lead to oxidative stress, cellular damage, and reduced physiological function, all of which accelerate aging; therefore, improved lifespan leads to improved lipid metabolism, which, in turn, may contribute to a reduction in fat accumulation [37]. The postbiotic not only improves the lifespan of the worms but also counteracts the negative effects of a high-fat diet. Remarkably, the postbiotic’s effect on longevity was significantly higher than that observed in the probiotic group, indicating that the postbiotic form is more effective in promoting healthspan. Additionally, the most impressive results came from the combinations of pA1c^®^HI with PC and Zn. Both combinations significantly increased the extension of life expectancy compared to pA1c^®^HI alone. The synergy observed between pA1c^®^HI and Zn or PC reinforces the idea that these bioactives may potentiate the effects of the postbiotic, leading to more pronounced benefits on longevity. Importantly, when zinc or chromium picolinate were used separately, no significant improvements were observed, further underscoring the need for a combined approach to achieve the observed effects. Similarly, oxidative stress also plays a crucial role in fat accumulation, as the buildup of ROS can lead to cellular damage and metabolic disruptions, promoting fat storage [38]. The significant reduction in ROS accumulation suggests that pA1c^®^HI effectively alleviates oxidative stress, a major contributor to metabolic dysfunction and aging. In particular, the postbiotic outperformed its probiotic counterpart, reinforcing the idea that heat inactivation can potentiate bioactive compounds responsible for antioxidant activity. These effects may be linked to the activation of key longevity-related pathways, such as *daf-16* and *skn-1*, both of which are upregulated following pA1c^®^HI supplementation. Given the established role of *daf-16* in lifespan extension and resistance to oxidative stress, its activation likely contributed to the observed improvements in survival and metabolic balance. Since life expectancy and oxidative stress are closely related to insulin resistance, lipid accumulation, and cellular damage, the ability of pA1c^®^HI to increase lifespan and mitigate ROS production highlights its potential for metabolic and anti-aging interventions.

Although our study provides promising evidence for the metabolic benefits of pA1c^®^HI, there are several limitations that should be addressed in future research. First, while *C. elegans* is a valuable model for understanding metabolic processes, further studies in higher organisms, including mammals, are necessary to confirm the translational relevance of these findings. Additionally, although the postbiotic pA1c^®^HI demonstrated superior efficacy over the probiotic form, the specific bioactive compounds responsible for these effects remain to be fully identified. Comprehensive metabolomic and proteomic profiling would help isolate and characterize the active compounds. Another area for improvement is the investigation of the long-term effects of pA1c^®^HI supplementation on metabolic health and potential side effects. Moreover, exploring the optimal dosing regimens and evaluating the interaction of pA1c^®^HI with other therapeutic agents could enhance its applicability in clinical settings. Importantly, the incorporation of postbiotics into food matrices represents a promising avenue for enhancing their therapeutic potential. Postbiotics are more stable than live probiotics, making them easier to integrate into various food products without the concerns associated with bacterial viability. Future research should focus on the development of strategies for incorporating postbiotics into food production processes, ensuring their stability during industrial processing, such as heat treatments, and their effectiveness when consumed as part of functional food products [39]. Understanding how postbiotics interact with different food ingredients and matrices could lead to new product formulations aimed at managing metabolic disorders like obesity, PreD, and T2D.

Altogether, these limitations offer clear directions for further research and development. Expanding this work into multi-omics systems biology approaches, validating in mammalian models, and evaluating efficacy in clinical trials will be necessary steps toward bringing postbiotics like pA1c^®^HI into functional nutrition and therapeutic settings.

## 4. Materials and Methods

### 4.1. Strains and Culture

*Pediococcus acidilactici* Lindner (Lactobacillales: Lactobacillaceae) strain was deposited, according to the Budapest Treaty, in the Spanish Collection of Type Cultures (CECT), with identification reference CECT 9879, from the proprietary strain collection of Genbioma Aplicaciones (Spain) backed by an international patent ‘Probiotics for regulating blood glucose [PCT/EP2020/087284; WO2021/123355A1]’. This species meets the criteria of qualified presumption of safety (QPS) by the European Food Safety Authority (EFSA). All postbiotic preparations were obtained by heat-inactivation of the corresponding *P. acidilactici* strain. The *C. elegans* strains used were N2 Bristol as wild-type strain, *daf-16* (mu86, CF1038) mutant strain, *daf-2*/*daf-16* (GR1308) double mutant strain, and *daf-16*:GFP mutant strain. *C. elegans* was cultured on NGM at 20 °C. *Escherichia coli* OP50 (*E. coli*, grown in LB Broth Lennox at 37 °C) was utilized as standard nematode sustenance.

### 4.2. C. elegans Experimental Design

All tests were carried out in quadruplicate in six-well cell culture plates with 4 mL of NGM or with 4 mL of glucose-loading (10 mM) NGM (NGMg) per well (with the pA1c^®^HI, the pA1c^®^, the chromium picolinate (PC), and the zinc spread or not inside the medium). Glucose was added to the medium with the aim of mimicking hypercaloric and obese conditions. Plates with orlistat (1.5 mg/mL, Sigma Aldrich, St. Louis, MO, USA) were used as a positive control of fat accumulation reduction based on previous studies where this drug for human use (pancreatic lipase inhibitor) was also used as positive control [40]. All experiments were performed at a concentration of 5 × 10^6^ cells/mL of pA1c^®^HI, 5 × 10^6^ CFU/mL of pA1c^®^, and 10 mM of PC and zinc. After pA1c^®^HI, pA1c^®^, PC, and zinc were added to NGM, the plates were left to harden and cool in the dark to prevent them from oxidizing in the presence of light. The heat treatments (121–135 °C) were performed directly on pA1c^®^HI before adding it to the plates. *E. coli* OP50 (150 µL of overnight culture) was seeded onto the plates, which were then dried again at room temperature in the dark. Age-synchronized worms were obtained through standard hypochlorite treatment. Eggs hatched overnight in M9 buffer, and approximately 2000 L1 larvae were transferred to the experimental plates and grown to the L4 or young adult stage [17].

### 4.3. Nile Red Staining

Nile Red staining is useful for the quantification of the fat accumulation of worms [41]. L4 worms grown under each condition were collected into 1.5 mL tubes, washed three times in PBST (PBS with 0.01% Triton X-100), chilled on ice for 15 min, and fixed in 40% isopropanol for 3 min. Nile Red solution (150 µL, 3 µg/mL; Thermo Fisher Scientific, Paisley, UK) was added, and samples were incubated for 30 min at room temperature with gentle shaking in the dark. Worms were washed in PBST and mounted on 2% agarose pads for microscopy [17].

### 4.4. Localization of daf-16 Gene

Synchronized L1 worms were grown on NGM or NGMg plates with or without pA1c^®^HI/pA1c^®^ supplementation for 46 h at 20 °C until reaching the L4 stage. Worms were anesthetized using 1% sodium azide in 2% agarose pads before imaging.

### 4.5. DHE Staining

Approximately 750 synchronized L1 larvae were transferred to the appropriate plates and grown to the L4 stage. Worms were harvested, washed in PBST, and incubated in 3 µM dihydroethidium (DHE; Sigma-Aldrich, Saint Louis, MO, USA) solution for 30 min. After washing, worms were mounted on agarose pads with sodium azide for imaging.

### 4.6. Image Acquisition and Quantification

Approximately 300 animals were analyzed per condition. Imaging was performed on a Leica stereomicroscope with standardized settings (calibration 0.68 µm/pixel, NA 0.15, resolution 1280 × 960, exposure 800 ms, analog gain 2.00, zoom 10×). GFP fluorescence was detected using Ex 480–500 nm, DM 505, BA 535–550 nm filters; DHE fluorescence with Ex 540–625 nm, DM 565, BA 605–655 nm; and daf-16 localization with Ex 340–380 nm, Em 435–485 nm filters. Quantification of fluorescence intensity, integrated density, and worm volume was performed using ImageJ v1.53e. For each condition, 25–40 worms were evaluated in four independent experiments.

### 4.7. Lifespan Analysis

L1 larvae were grown to L4, and then 50–65 individuals per replicate (*n* = 4) were transferred to fresh plates containing the same treatment plus 40 µM 5-fluoro-2-deoxyuridine (FUDR #856657, Sigma-Aldrich, St. Louis, MO, USA) to prevent progeny. Worms were scored daily as alive or dead (no response to gentle touch) until all had died.

### 4.8. Egg Laying and Development Analysis

To determine whether treatments affected reproduction or development, egg laying was assessed in young adults (day 3). Worms were grown on NGMg plates with or without pA1c^®^HI/pA1c^®^, and images were taken in situ using a Nikon SMZ18 stereomicroscope equipped with a DS-Fi1C camera at 135× magnification (Nikon Instruments Inc., Tokyo, Japan).

### 4.9. RNA Extraction and Quantitative PCR Analyses

Eight biological replicates of synchronized L1 worms were grown on NGM or NGMg plates. Total RNA was extracted using Trizol^®^ reagent (Thermo Fisher Scientific, Paisley, UK) and quantified using a NanoDrop ND-1000 spectrophotometer (Thermo Fisher Scientific, Wilmington, DE, USA). RNA (1 g) was treated with DNase I (AmbionTM DNase I, RNase-free; Thermo Fisher Scientific Inc., Waltham, MA, USA) and reverse-transcribed into cDNA. Gene expression was quantified by qPCR using TaqMan Universal Master Mix and gene-specific probes (Thermo Fisher Scientific Inc., Waltham, MA, USA) and Integrated DNA Technologies Inc. (Coralville, IA, USA) on a CFX384 Touch Real-Time PCR System (BioRad, Hercules, CA, USA). The *pmp-3* gene (TaqMan Gene Expression Assays, Carlsbad, CA, USA) served as an internal reference, and relative expression of the measured gene was calculated using the 2^−ΔΔCt^ method.

### 4.10. Statistical Analysis

Differences in fat storage (Nile Red), ROS levels (DHE), and gene expression were analyzed by one-way ANOVA followed by the Student–Newman–Keuls (SNK) post-hoc test when *p* < 0.05. Lifespan curves were compared using the log-rank (Mantel–Cox) test. All analyses were performed with GraphPad Prism v9.

## 5. Conclusions

Based on our results, pA1c^®^HI demonstrates significant potential as an interventive strategy for improving metabolic health by modulating key pathways involved in glucose and lipid metabolism. Our study highlights its superior efficacy over its probiotic counterpart in enhancing glucose metabolism and insulin signaling, promoting fatty acid β-oxidation, and reducing fatty acid biosynthesis, as evidenced by changes in gene expression. The synergistic effects observed when combining pA1c^®^HI with bioactive compounds like Zn and PC further improve its metabolic benefits, suggesting its potential for wider applications in managing PreD, T2D, and obesity. Additionally, the stability and ease of incorporation of the postbiotic ingredient pA1c^®^HI into food production processes make it a promising candidate for the development of functional foods aimed at enhancing metabolic health.

We acknowledge the inherent limitations of the *C. elegans* model in fully replicating mammalian metabolic complexity. Therefore, future studies will focus on validating these promising findings in murine models and human in vitro systems to better understand the translational potential of pA1c^®^HI. These steps are essential to confirm its efficacy and safety before potential clinical applications.

Overall, our results firmly establish pA1c^®^HI as a highly effective and stable postbiotic, offering distinct advantages over live probiotics in the management of metabolic disorders, making it a robust candidate for food and therapeutic applications.

## Data Availability

The original contributions presented in this study are included in the article. Further inquiries can be directed to the corresponding author.
